# Prevalence, Species Characterization, and Genetic Diversity of *Bartonella* Infections in Rodents From Mudflat Wetlands Along the Eastern Coast of Jiangsu Province in China

**DOI:** 10.1155/jotm/9926259

**Published:** 2025-04-23

**Authors:** Chen Guoqing, Li Chunxiang, Cui Qian, Li Changcheng, Yang Pengfei, Yan Qingli, An Ran, Liu Wei, Li Feng, Lu Kuikui, Zhang Hongjun, Peng Haiyan

**Affiliations:** ^1^Department of Pathogenic Microbiology and Biological Laboratory, Yancheng Municipal Center for Disease Control and Prevention, Yancheng, Jiangsu, China; ^2^Laboratory Department, Yancheng Maternal and Child Health Hospital, Yancheng, Jiangsu, China; ^3^Department of Disinfection and Vector Biology Control, Yancheng Municipal Center for Disease Control and Prevention, Yancheng, Jiangsu, China; ^4^Department of Acute Infectious Disease Prevention and Control (Office of Health Emergency), Yancheng Municipal Center for Disease Control and Prevention, Yancheng, Jiangsu, China; ^5^Laboratory Department, Huai'an Municipal Center for Disease Control and Prevention, Huai'an, Jiangsu, China; ^6^Department of Comprehensive Business and Quality Control Management, Yancheng Municipal Center for Disease Control and Prevention, Yancheng, Jiangsu, China; ^7^Development Zone Branch, Yancheng Municipal Center for Disease Control and Prevention, Yancheng, Jiangsu, China; ^8^Institute of Toxicology and Risk Assessment, Jiangsu Provincial Center for Disease Control and Prevention, Nanjing, Jiangsu, China; ^9^Central Office, Yancheng Municipal Center for Disease Control and Prevention, Yancheng, Jiangsu, China; ^10^Editorial Department, Jiangsu Provincial Center for Disease Control and Prevention, Nanjing, Jiangsu, China

**Keywords:** 16S rRNA gene, *Bartonella*, *gltA* gene, PCR, phylogenetic analysis, rodent

## Abstract

**Objective:** To investigate the infection status, species composition, and genetic diversity of *Bartonella* in local rodent populations in coastal mudflat wetland habitats in eastern Jiangsu Province of China.

**Methods:** From March to June 2023, rodents were captured in mudflat wetlands of Dongtai and Tinghu Counties, Eastern China. Rodent species were identified, and nucleic acids were extracted from liver and spleen tissues. The mitochondrial cytochrome b (mt-*cytb*) gene was amplified by PCR, while *Bartonella*-specific citrate synthase (*gltA*) and 16S rRNA genes were amplified by semi-nested PCR. Phylogenetic and homology analyses were conducted to identify rodent and *Bartonella* species.

**Results:** Among 29 captured rodents, 26 were *Apodemus agrarius* and 3 were *Mus musculus*. Phylogenetic analysis of the mt-*cytb* gene divided *A. agrarius* into 7 lineages, each linked to geographically diverse *Bartonella* populations. Six *A. agrarius* rodents tested positive for *Bartonella*, with a positivity rate of 20.69%. Phylogenetic analyses revealed three *Bartonella* species: *B. fuyuanensis*, *B. taylorii*, and one undetermined species. The infected *Bartonella* strains clustered into three evolutionary branches based on *gltA* and 16S rRNA genes.

**Conclusions:** This study provides the first evidence of *Bartonella* infection among rodent populations in wetland habitats along China's eastern coast. The region harbors diverse rodent species, with a high *Bartonella* infection rate, and at least three species were identified, including a potential novel species.

## 1. Background

Bartonellosis, caused by intracellular, Gram-negative bacteria of the genus *Bartonella*, is a vector-borne, hemotrophic, zoonotic infectious disease [1, 2]. To date, over 50 species of *Bartonella* have been identified, more than 10 of which are known to cause human diseases such as endocarditis, myocarditis, meningitis, tick-associated swelling, and cat scratch disease [1, 3, 4]. Transmission to humans occurs either indirectly through hematophagous arthropods or directly via contact with infected cats, dogs, or contaminated feces [5, 6]. *Bartonella* has a broad range of natural reservoirs, with rodents being recognized as significant hosts [7–9]. Therefore, understanding the prevalence and genetic characteristics of *Bartonella* in rodent populations is crucial for evaluating transmission risks to humans and wildlife.

The mitochondrial cytochrome b (mt-*cytb*) gene is a widely used molecular marker for analyzing population evolution, genetic structure, and geographical differentiation, aiding in species classification and phylogenetic studies [10, 11]. Additionally, the 16S rRNA and citrate synthase (*gltA*) genes are commonly utilized for species identification, as well as for genetic diversity and phylogenetic analysis of *Bartonella* [5, 12]. In this study, we analyzed all three molecular markers—mt-*cytb*, 16S rRNA, and *gltA*—to explore rodent population evolution, classify *Bartonella* species, and assess their phylogenetic relationships and genetic diversity.

Yancheng City, located in Jiangsu Province on the eastern coast of China, is home to the largest muddy intertidal zone and wetland habitat along the Western Pacific. The diverse landforms foster a wide variety of vector organisms, and the geographical environment is highly conducive to rodent populations. Human activity in these natural epidemic areas disrupts the ecological balance, heightening the public health risks of zoonotic diseases like bartonellosis. To date, no studies have reported on local *Bartonella* infections. The objectives of this study were to establish a methodology and conduct a preliminary investigation in two typical surveillance sites, thereby providing a scientific basis for the development of prevention and control strategies for humans, domestic animals, and wildlife. This study also aims to create a foundational profile of the prevalence, species composition, and genetic diversity of *Bartonella*-carrying rodents in the mudflat and wetland habitats along China's eastern coast.

## 2. Materials and Methods

### 2.1. Rodent Sample Collection

**Figure 1:**
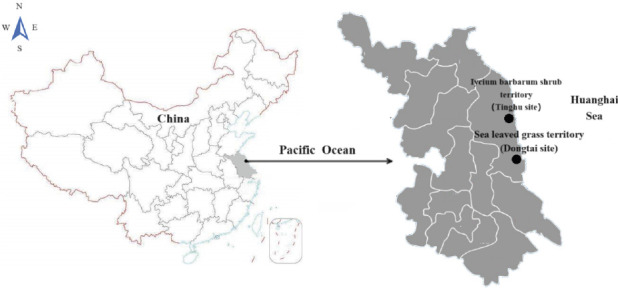
Geographical locations of the rodent sampling sites (black dots) in the mudflat wetlands along the eastern coastal areas of China. The map was generated using Epi Map software.

From March to June 2023, rodents were captured monthly from two surveillance sites within the mudflat wetland habitats of Dongtai Tiaozini Scenic Area (120°85′83″–120°89′50″ E, 32°74′09″–32°74′12″ N, the Dongtai site), representing a sea-leaved grass territory, and the Tinghu Red-Crowned Crane Nature Reserve (120°52′86″–120°85′83″ E, 33°31′38″–33°74′09″ N, the Tinghu site), representing a *Lycium barbarum* shrub territory, both located in Yancheng City, Jiangsu Province, on the eastern coast of China. Rodents were trapped over four months using the overnight cage method, with 120 cages set up at each site at sunset and baited with deep-fried dough sticks or ham sausage. The traps were collected the next morning. Captured rodents were placed in rat bags, relevant information was recorded, and samples were immediately sent to the laboratory for analysis (Figure 1). This project was reviewed and approved by the Animal Ethics Committee of Jiangsu Provincial Center of Disease Control and Prevention.

### 2.2. Morphological Identification of Rodent Species

The captured rodents were identified to species and gender based on morphological characteristics, including appearance, skull, teeth, and other relevant features. Liver and spleen samples were collected under sterile conditions and stored at −80°C for further analysis.

### 2.3. DNA Extraction and PCR Detection of Rodent and *Bartonella* Genes

The captured rodents were placed in a transparent, sealed plastic box along with ether soaked cotton balls for anesthesia; all efforts were conducted in order to minimize pain. The captured animals were euthanized by cervical dislocation under deep anesthesia, and the liver and spleen tissues of the rodents were collected under sterile conditions and stored at −80°C for later analysis. Liver and spleen tissues were collected from the rodents and placed into 1.5 mL microcentrifuge tubes in a biosafety cabinet. Lysis buffer was added, and the tissues were shredded and homogenized. DNA was extracted using the TQ-BG-001-200 kit (Shanghai Bojie Medical Technology Co., Ltd.), serving as a template for detecting the mt-*cytb*, *gltA*, and 16S rRNA genes with PowerPol 2× PCR Mix (AB clonal Cat: RK20719, Wuhan Aibotec Biotechnology Co., Ltd.).

The Cytb gene (1140 bp) was amplified via standard PCR with the primers Cytb-F (CGAAGCTTGATAYGAAAAAYCYGYGTTG) and Cytb-R (TAGAATAYCAGCTTTGGGTG). The *gltA* (476 bp) and 16S rRNA (813 bp) genes were amplified using a semi-nested PCR method [5].

### 2.4. Nucleotide Sequence Analysis

PCR products for the mt-*cytb*, *gltA*, and 16S rRNA genes were purified and sequenced by Shanghai Shenggong Company. The resulting sequences were searched against the GenBank database using BLAST to identify rodent and *Bartonella* species. The nucleotide identities of *gltA* and 16S rRNA were calculated using the MegAlign program in DNASTAR Lasergene software [13]. Phylogenetic trees were constructed using PhyML 3.0 software, employing the maximum likelihood (ML) method [14]. The best-fit nucleotide substitution model (GTR + Γ + I) for phylogenetic analysis was determined using MEGA 6.0.6 [15]. Bootstrap analysis with 1000 replicates was performed to assess tree reliability, with a threshold of ≥ 96% homology for defining phylogroups [16].

### 2.5. Statistical Analysis

Statistical analyses were conducted using SPSS 16.0 software. Chi-square (*χ*^2^) tests were used to compare the positive rates of *Bartonella* infection among different rodent species and genders. A *p* value of < 0.05 was considered statistically significant.

## 3. Results

### 3.1. Rodent Species and Composition

A total of 29 rodents were captured from the two surveillance sites, with 23 collected at the Dongtai site (79.31%) and 6 at the Tinghu site (20.69%). Males made up the majority of the sample (16, accounting for 55.17%). Morphological identification revealed that the rodents belonged to the order Rodentia, family *Muridae*, with two species identified: *Apodemus agrarius* and *Mus musculus*. The dominant species was *A. agrarius*, which accounted for 89.66% (26/29) of the total sample, comprising 14 males (53.85%) and 12 females (46.15%). The remaining 3 rodents (10.34%) were *M. musculus*, with 2 males (66.67%) and 1 female (33.33%) (Table 1).

### 3.2. Phylogenetic Analysis of Rodent Population Based on the mt-*cytb* Gene

**Figure 2:**
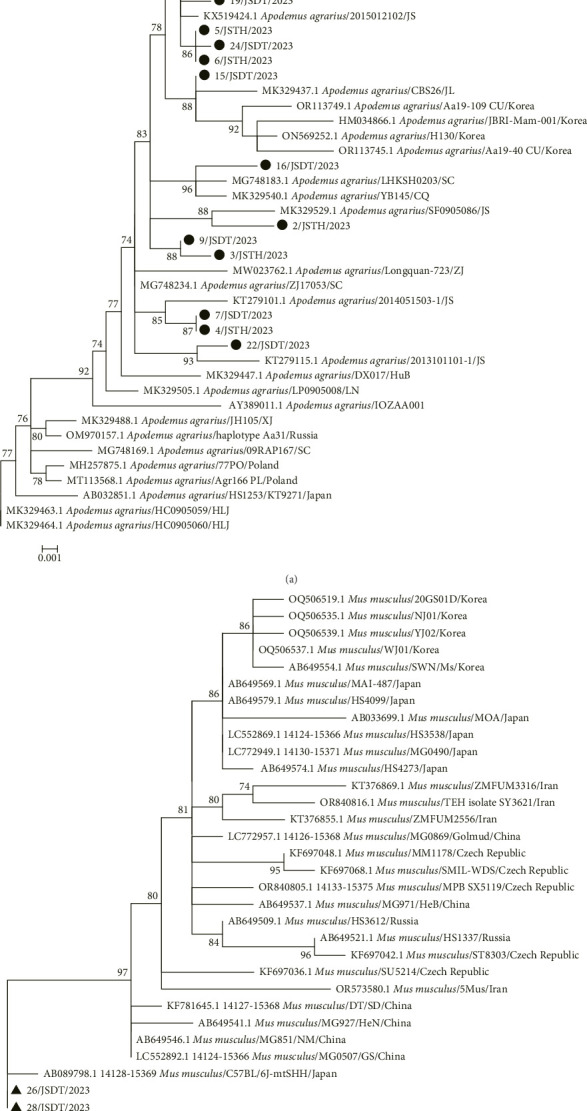
Phylogenetic tree of *A. agrarius* (a) and *M. musculus* (b) from the two sampling sites. Bootstrap values were calculated using 1000 replicates, with only values > 70% shown. Sequences determined in this study are marked with a black circle (● for *A. agrarius*) and a black triangle (▲ for *M. musculus*). The scale bar represents the number of nucleotide substitutions per site.

The mt-*cytb* gene was successfully amplified and sequenced from 23 rodents, including 21 *A. agrarius* rodents and 2 *M. musculus* rodents. A phylogenetic tree was constructed using mt-*cytb* gene sequences from these rodents, along with representative geographic populations of *A. agrarius* and *M. musculus* from the GenBank database. The results showed that *A. agrarius* from the two sampling sites was divided into seven evolutionary branches. The dominant branch consisted of 13 strains (1/JSTH/2023, 5/JSTH/2023, 6/JSTH/2023, 11/JSDT/2023, 12/JSDT/2023, 13/JSDT/2023, 15/JSDT/2023, 17/JSDT/2023, 18/JSDT/2023, 19/JSDT/2023, 21/JSDT/2023, 23/JSDT/2023, and 25/JSDT/2023), which clustered with representative strains from Jiangsu Province (KT279107 and KX519424). The remaining 6 strains (2/JSTH/2023, 4/JSTH/2023, 7/JSDT/2023, 15/JSDT/2023, 16/JSDT/2023, and 22/JSDT/2023) clustered with strains closely related to their domestic relatives, forming five scattered evolutionary branches. Strains 3/JSTH/2023 and 9/JSDT/2023 formed independent branches. Other domestic representative strains, such as the Xinjiang strain (MK329488), and foreign representative strains, including the South Korean strain (OR113745), Japanese strain (AB032851), Russian strain (OM970157), and Polish strain (MH257875), showed distant phylogenetic relationships and did not cluster into any specific evolutionary branch. Strains 26/JSDT/2023 and 28/JSDT/2023, which clustered with rodents from Dongtai County, showed similar phylogenetic relationships to the Japanese strain (AB089798) and belonged to the same evolutionary branch. However, these two strains had distant relationships with other representative strains from China and abroad and did not cluster within the same evolutionary branches (Figure 2).

### 3.3. PCR Detection Results of *Bartonella gltA* and 16S rRNA Genes

A total of 58 liver and spleen tissue samples from 29 rodents were tested for the *Bartonella gltA* and 16S rRNA genes. Six *A. agrarius* individuals showed positive PCR results for both liver and spleen samples, with an overall *Bartonella* positive rate of 20.69%, and a positive rate of 23.08% among *A. agrarius*. Four *A. agrarius* from Dongtai and two from Tinghu tested positive, resulting in positive rates of 17.39% (4/23) and 33.33% (2/6), respectively. There was no statistically significant difference in detection rates between the two sites (*χ*^2^ = 0.086, *p* > 0.05).

### 3.4. Nucleotide Homology Analysis of *Bartonella gltA* and 16S rRNA Genes

The 12 valid PCR products from the six positive *A. agrarius* were sequenced for *gltA* and 16S rRNA genes. The sequences from each individual's liver and spleen samples were identical, with no evidence of multiple infections, missing nucleotides, or insertions. Homology analysis revealed nucleotide similarities between strains 3/JSTH-Aa/2023, 5/JSTH-Aa/2023, and 15/JSDT-Aa/2023 and the *B. fuyuanensis* strain DSM100694 (NZ JACIFE010000010), ranging from 99.8% to 100% for the *gltA* gene and 100% for the 16S rRNA gene. The *gltA* gene of strain 24/JSDT-Aa/2023 had a 99.6% similarity to the *B. taylorii* strain Chengdu Rn40 (OP382433), while its 16S rRNA gene had 100% similarity to the *B. taylorii* strain IBS296 (CP083444). The *gltA* gene of strains 17/JSDT-Aa/2023 and 19/JSDT-Aa/2023 showed 100% similarity to the uncultured strain *B*. sp. clone 66 (OP022209). However, these strains had a maximum *gltA* similarity of 94.9% with *B. krasnovii* strain D1 (MH618787), and their highest 16S rRNA similarity was 99.3% with *B. queenslandensis* strain NH5 (EU111755).

### 3.5. Species Identification and Phylogenetic Analysis of *Bartonella gltA* and 16S rRNA Genes

**Figure 3:**
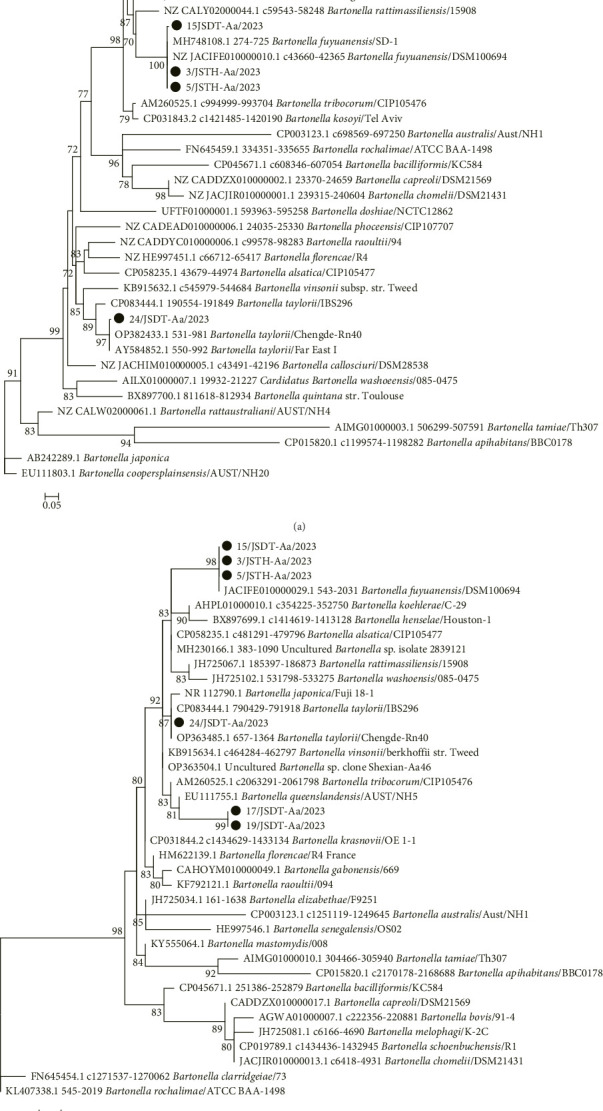
Phylogenetic trees of *Bartonella* based on (a) *gltA* and (b) 16S rRNA genes. Bootstrap values were calculated with 1000 replicates and only > 70% are shown. Sequences determined in this study are marked with a black circle, and the scale bar represents the number of nucleotide substitutions per site.

The six positive rodents were identified as carriers of 3 types of *Bartonella*: *B. fuyuanensis*, *B. taylorii*, and an undetermined species (Figure 3). Reference strains from various *Bartonella* species in the GenBank database were used alongside the sequences obtained in this study to generate phylogenetic trees based on *gltA* and 16S rRNA genes. The phylogenetic trees displayed similar topological structures, with the *Bartonella* from the 6 rodents forming three evolutionary branches: 3/JSTH-Aa/2023, 5/JSTH-Aa/2023, and 15/JSDT-Aa/2023 clustered with the *B. fuyuanensis* strain DSM100694 (NZ JACIFE010000010) to form the *B. fuyuanensis* branch; 24/JSDT-Aa/2023 clustered with *B. taylorii* strains Chengdu Rn40 (OP382433) and IBS296 (CP083444) to form the *B. taylorii* branch; and 17/JSDT-Aa/2023 and 19/JSDT-Aa/2023 formed an independent undetermined branch.

## 4. Discussion

*Bartonella* infection can cause acute and chronic systemic multiorgan diseases in humans, with severe cases potentially being fatal [17, 18]. According to both domestic and international studies [19–21], rodents exhibit a high infection rate of *Bartonella*, with considerable genetic diversity, which indicates a significant risk of zoonotic transmission to humans and animals. Cases of *Bartonella* infection have been documented in countries such as the United States [22], Ethiopia [23], Thailand [24], and China [25]. Although no human infections have been reported in Yancheng City, the habitat's suitability for rodent populations necessitates the evaluation of potential risks. Vector-borne diseases, including *Bartonella*, should be monitored in local rodents to assess the risk of transmission.

In this study, 29 rodents were collected from two coastal wetland sampling sites. Only 2 species of *A. agrarius* and *M. musculus* were identified, with *A. agrarius* being the dominant species, accounting for 79.31% of the total rodents collected, particularly at the Dongtai site. The higher rodent density in Dongtai could be attributed to the favorable riverbank grassland habitat, whereas the Tinghu Red-Crowned Crane Reserve, characterized by *Lycium barbarum* shrubland and tourist activities, may offer less suitable conditions for rodent proliferation.

The phylogenetic analysis of mt-*cytb* gene lineages from different geographical populations of *A. agrarius* and *M. musculus* revealed genetic diversity among local rodent populations. *A. agrarius* was divided into 7 evolutionary branches across the 2 sites. Six of these branches were closely related to other rodent populations in China, suggesting gene flow and evolutionary cycling among geographical populations. Strains 3/JSTH/2023 and 9/JSDT/2023, however, formed independent branches, indicating unique genetic origins distinct from both domestic and foreign strains. Additionally, *M. musculus* strains 26/JSDT/2023 and 28/JSDT/2023 clustered with the Japanese strain (AB089798), forming an independent evolutionary branch, suggesting potential genetic exchange via certain vectors.

The *gltA* and 16S rRNA genes of *Bartonella* were sequenced for further analysis, and both genes displayed highly similar clustering relationships, providing a robust molecular foundation for future surveillance efforts. Among the 29 rodent liver and spleen samples tested for *Bartonella* infection, six *A. agrarius* samples were positive, resulting in a nucleic acid positivity rate of 20.69%. This rate is higher than those reported in Guangzhou (6.4%) [26], Guizhou (16.1%) [27], Hebei (17%) [5], and Southeastern China (14.9%) [28], but lower than those reported in Shanxi (26.1%) [4], the Qinghai-Tibet Plateau (30.1%) [29], Zhongtiao Mountain (49.5%) [30], and Heixiazi Island (57.7%) [31]. The positivity rate is also comparable to that found in Japan, Korea, and Thailand (28.8%–56.30%) [32].

Phylogenetic analysis of the *gltA* and 16S rRNA genes identified 3 species of *Bartonella: B. fuyuanensis*, *B. taylorii*, and an undetermined species within the six positive *A. agrarius* rodents. Strains 17/JSDT-Aa/2023 and 19/JSDT-Aa/2023 formed an independent evolutionary branch, distinct from known species in the GenBank database. These strains shared the highest nucleotide similarity (94.9%) with *B. krasnovii* strain D1 (MH618787) for the *gltA* gene and 99.3% similarity with *B. queenslandensis* strain NH5 (EU111755) for the 16S rRNA gene, suggesting the possibility of a new *Bartonella* species. The conservation of the 16S rRNA gene, compared to the more variable *gltA* gene, supports this conclusion.

This is the first study to identify *Bartonella* in local rodents on the eastern coast of Jiangsu Province, indicating the potential risk of interspecies transmission to humans or other wildlife. The local *Bartonella* infections in rodents were mainly detected in *A. agrarius*, which expanded the types as well as the geographical distribution of the *Bartonella* infections in rodents in local area. However, in this study, only the rodents were captured, followed by detection of the *Bartonella* to provide the possibility of the infection identification in local rodent population; no ectoparasites of ticks, lice, or fleas attached to the captured rodents were harvested, and thus whether the attached ectoparasites could be infected with the *Bartonella* or whether the rodents should share the same types of the *Bartonella* with the ectoparasites still remained unrevealed. The rodents commonly serve as reservoirs for numerous vector-borne pathogens, including *Rickettsia*, *Leptospira*, *Yersinia pestis*, rabies virus, and coronaviruses. Thus, comprehensive surveillance of rodent populations is critical for understanding the prevalence and pathogen spectrum in order to evaluate the risks of possible spreading of zoonotic diseases, which will require ectoparasite collection and analysis in the future.

In summary, this study represents a pioneering effort to investigate rodent populations and *Bartonella* species in the classical muddy intertidal zone and wetland habitats of eastern Jiangsu Province. The identification of diverse rodent and *Bartonella* species suggests a potential risk for the interspecies transmission of vector-borne diseases. From a genetic perspective, the presence of similar genetic flow among different rodent populations could facilitate the spread of *Bartonella* across geographical regions. However, future research should expand the sampling sites, increase the number of rodents, and extend the sampling duration. It should also consider ecological factors such as the rodents' growth cycles, habitat distribution, and ecological communities to paint a more comprehensive picture of rodent and *Bartonella* distribution. This study provides a foundation for future surveillance efforts, contributing to a better understanding of the local rodent population and the pathogens they carry.

## Figures and Tables

**Table 1:** The composition of captured rodents in 2 sampling sites (*n*).

Rodent species	Dongtai site			Tinghu site			Total		
	F	M	Total	F	M	Total	F	M	Total
*A. agrarius*	11	9	20	3	3	6	14	12	26
*M. musculus*	2	1	3	0	0	0	2	1	3
Total	13	10	23	3	3	6	16	13	29

## Data Availability

The data that support the findings of this study are available from the corresponding author upon reasonable request.
